# Splenic CD11c^low^CD45RB^high^ dendritic cells derived from endotoxin-tolerant mice attenuate experimental acute liver failure

**DOI:** 10.1038/srep33206

**Published:** 2016-09-14

**Authors:** Sai-Nan Zhang, Nai-Bin Yang, Shun-Lan Ni, Jin-Zhong Dong, Chun-Wei Shi, Shan-Shan Li, Sheng-Guo Zhang, Xin-Yue Tang, Ming-Qin Lu

**Affiliations:** 1 Department of Infection Diseases, The First Affiliated Hospital of Wenzhou Medical University, Wenzhou Key Laboratory of Hepatology, Hepatology Institute of Wenzhou Medical University, Wenzhou 325000, Zhejiang, P. R. China; 2 Department of Intensive Care Unit, The First Hospital of Ningbo, Ningbo 315010, Zhejiang, P. R. China; 3 Department of Infection Diseases, The First Hospital of Xiaoshan, Hangzhou 311200, Zhejiang, P. R. China

## Abstract

Endotoxin tolerance (ET) is suggested to attenuate the severity of acute liver failure (ALF) in mice, possibly through both innate and adaptive immunity. However, the involvement of regulatory dendritic cells (DCregs) in ET has not been fully elucidated. In this study, their effect on ALF in mice was investigated. Splenic DCregs from ET-exposed mice (ET-DCregs) showed lower expression levels of CD40, CD80, and MHC-II markers and stronger inhibition of allogenic T cells and regulation of IL-10 and IL-12 secretion than splenic DCregs from normal mice (nDCregs). Moreover, the mRNA and protein levels of TNF-α and P65 in splenic ET-DCregs were significantly lower than those in the splenic nDCregs. The survival rate was significantly increased and liver injury was mitigated in mice with ALF treated with splenic ET-DCregs. In addition, A20 expression was decreased in the liver of ALF mice, but elevated after infusion of splenic nDCregs and ET-DCregs, and a much higher elevation was observed after infusing the latter cells. The functionality of splenic DCregs was altered after ET exposure, contributing to protection of the livers against D-GalN/LPS-induced ALF.

Acute liver failure (ALF) is a life-threatening syndrome primarily caused by severe liver injury. Although the etiologies of ALF are diverse and the pathogenesis is still yet to be fully elucidated, disruption of the immune response has been reported upon ALF. Several animal models of ALF have been generated that can recapture the clinical phenotype and course of ALF^1^. For example, ALF can be induced by infusion of lipopolysaccharide (LPS)/D-galactosamine (D-GalN). In addition, we and others have demonstrated that pre-treatment of animals with a small amount of LPS attenuates the severity of D-GalN/LPS induced ALF (endotoxin tolerance, ET), and the mechanism responsible for this protection is related to immune modulation of LPS through TLR4 signaling^2^.

Although it is well known that innate immune cells like monocytes/macrophages, when exposed to a small amount of endotoxin, become endotoxin tolerant, the impact of exposure on other immune cells such as dendritic cells (DCs) in the setting of ET remains unclear. DCs were first identified from the skin and named by Dr. Langerhans in 1868 because of their distinct morphological features with dendrites. Later studies revealed that DCs are one of the most important antigen-presenting cells (APCs)^3^,^4^. DCs circulate in peripheral blood and reside in lymphoid tissues. The DCs in peripheral blood account for less than 1% of all peripheral blood mononuclear cells, but they are essential for bridging the innate and adaptive immune systems. DCs function to launch and coordinate appropriate immune responses to pathogens and to prime memory of immune cells so that the host will be under protection upon future infections^4^. As an early step of initiating an adaptive immune response to pathogens, DCs uptake, process, and present antigens to T cells^5^,^6^,^7^. The antigen presenting function of DCs is closely related to the expression of marker proteins on cellular surface including major histocompatibility complex class II (MHC-II) and costimulatory molecules CD80 and CD86.

DCs can be classified into different subsets in line with their functions and/or developmental stages. The splenic DC class contains heterogeneous subsets of DCs with a spectrum of functions and morphologies^8^. One subset of DCs is regulatory DCs (DCregs), which modulate immune cells and contribute to the host immune tolerance by maintaining immune response at steady state. CD11C^low^CD45RB^high^ DCs, a subtype of DCregs with low expression of CD11c and high expression of CD45RB, can respond to an inflammatory stimulus^9^. Recent studies also suggested that splenic DCregs inhibit inflammation by generating anergic T cells and regulatory T cells (Tregs), as well as deleting peripheral T cells under lethal conditions of endotoxemia and bacterial peritonitis^10^. Therefore, in the current study, we examined the effect of splenic DCs derived from LPS-pretreated mice on D-GalN/LPS–induced acute liver failure and explored the possible mechanisms underlying ET.

## Material and Methods

### Ethics statement

This research project including survival analysis was approved by the Institute Animal Care and Use committee at Wenzhou Medical University (Approval Number: wydw2012-0053).

### Animal experiment

Male BALB/c mice aged 6–8 weeks were purchased from the Shanghai Laboratory Animal Center (Shanghai, China). All mice were caged in specific pathogen-free rooms in the Experimental Animal Laboratory of Wenzhou Medical University (Wenzhou, Zhejiang, China) under standard laboratory conditions (laminar-flow, temperature-controlled by 21 ± 2 °C, humidity-regulated at 40–70%, 12-h light/dark cycle), fed with a standard chow diet and free water and treated in accordance with the Guide for the Care and Use of Laboratory Animals of the National Institutes of Health.

### Experimental design

D-GalN (Sigma-Aldrich, St. Louis, MO, USA) and LPS (Sigma-Aldrich) were dissolved in sterile 0.9% sodium chloride (NaCl). Mice were randomly divided into four groups: control group (n = 15), ALF group (n = 20), nDCregs group (n = 20), and ET-DCregs group (n = 20). To establish the experimental model of ALF, all mice in the ALF, nDCregs, and ET-DCregs groups were intraperitoneally injected with 500 μL of sterile 0.9% NaCl containing 20 mg D-GalN and 10 μg/kg LPS. In control group, mice were intraperitoneally injected with 500 μL of sterile 0.9% saline. After 30 minutes of D-GalN/LPS injection, nDCregs (10^6^/mouse, 200 μl, i.p.) were infused into mice in the nDCregs group and ET-DCregs (10^6^/mouse, 200 μl, i.p.) were administered into mice in the ET-DCregs group. As a control, mice in the ALF group were injected with 200 μl of sterile 0.9% saline. Three mice were sacrificed with chloral hydrate at time points of 2, 6, 12, 24, and 48 hours after D-GalN/LPS injection. Both serum and liver samples were collected and stored at −80 °C for further analysis. A portion of each liver specimen was fixed in 10% neutral formalin for histopathological analysis. In a separate experiment, the 7-day mortality in another 40 mice consisting of control (n = 10), ALF (n = 10), nDCregs (n = 10), and ET-DCregs (n = 10) groups were monitored once every 6 hours after D-GalN/LPS injection without euthanasia before the experimental endpoint. The aim of the 7-day survival analysis was to investigate the possible improvement by transferring splenic CD11c^low^CD45RB^high^ DCs derived from endotoxin-tolerant mice in the survival rate of ALF mice. The observation for death after intraperitoneal injection of D-GalN/LPS with or without subsequent administration of special DCs was the part of the approved protocol.

### DCreg isolation and culture

Male BALB/c mice were randomly divided into normal and ET groups. LPS was intraperitoneally injected into ET mice at 0.1 mg/kg daily for 5 days, and the same volume of sterile 0.9% sodium chloride was injected into mice in the normal control group. All mice were euthanized with chloral hydrate (1.0 g/kg, i.p.) for harvesting of the spleen tissues 5 days later. Spleens were then cut into small pieces, and cells were dissociated with collagenase D (Roche, Basel, Switzerland) digestion under aseptic conditions. After inhibition of collagenase with 10 mM EDTA (ethylenediaminetetraacetic acid), cells were resuspended in phosphate-buffered saline (PBS) containing 2 mM EDTA and 2% fetal calf serum (FCS, Gibco, Grand Island, NY, USA). Single-cell suspensions were prepared using the standard procedure. Erythrocytes were removed by osmotic lysis. Splenic CD11c^low^CD45RB^high^ DCs were enriched by negative selection with anti-mouse CD11c monoclonal antibody (mAb) magnetic beads and a MACS column (eBioscience, San Diego, CA, USA). These CD11c^low^ DCs were then poured into a MACS column with anti-mouse CD45RB mAb magnetic beads to isolate CD45RB^high^ DCs. More than 90% of purified cells were CD11c^low^CD45RB^high^. Splenic DCregs were finally washed and suspended in RPMI-1640 (Gibco) supplemented with 10% FCS. A portion of splenic DCregs was prepared for observation of cell morphology. The final concentration of splenic CD11c^low^CD45RB^high^ DCs from normal (n-DCregs) and LPS-pretreated mice (ET-DCregs) was adjusted to 1 × 10^5^/ml. The 10 ml of CD11c^low^CD45RB^high^ DC suspension at final concentration of 1 × 10^5^/ml were centrifuged, the supernatant was removed, and these DCs were resuspended with 0.2 ml saline, which was intraperitoneally injected into each mouse.

CD4^+^ T cells from normal BALB/c mice were purified from single splenocyte suspensions using the murine CD4^+^ T-cell isolation kit and MACS magnetic columns according to the manufacturers’ instructions. Isolated CD4^+^ T cells (2 × 10^6^/ml, >90% purity) in complete RPMI-1640 (Gibco) were co-cultured with purified splenic DCregs at various ratios in 96-well plates in an incubator supplied with 5% CO_2_ and kept at 37 °C. A CD4^+^ T-cell culture without DCregs was used as the control group. After 24 hours of co-culture, CD4^+^ T cells were separated from splenic DCregs using the murine CD4+ T-cell isolation kit and MACS magnetics columns and tested for suppressive activity using the Cell Counting kit-8 (CCK-8) (Donjindo Laboratories, Kumamoto, Kyushu, Japan). Meanwhile, cell culture supernatants were collected and analyzed for cytokine levels using antibody enzyme-linked immunosorbent assay (ELISA) kits per the manufacturers’ specifications.

### Flow cytometry

A flow cytometry (FACSCalibur flow cytometer, Becton Dickinson Immunocytometry Systems, San Jose, CA, USA) was used to assess the expression levels of adhesive and co-stimulatory molecules on splenic DCregs isolated from normal mice and mice with ET. Cells were stained with fluorescein-conjugated mAbs to mouse CD11c, CD45RB, CD40, CD80, and MHC-II (all from eBioscience). The acquired data were processed using FlowJo Software (TreeStar, Palo Alto, CA, USA).

### ELISA

Cytokines in cell culture supernatants (interleukin [IL]-10 and IL-12) and serum (IL-6 and tumor necrosis factor [TNF]-α) were measured with ELISA kits (Beyotime Institute of Biotechnology, Nanjing, China).

### Real-time PCR

Total RNA was extracted from liver tissue using RNAiso Plus reagent (Aidlab Biotechnologies Co., Beijing, China). Reverse transcription was conducted using SYBR ExScript RT-PCR kit (Aidlab Biotechnologies Co.). The primers for target genes were as follows: A20 (224 bp), forward: 5′-AAACCAATGGTGATGGAAACTG-3′ and reverse: 5′-CTTGTCCCATTCGTCATTCC-3′; TNF-α (111 bp), forward: 5′-CTACTCCCAGGTTCTCTTCAA-3′ and reverse: 5′-GCAGAGAGGAGGTTGACTTTC-3′; P65 (235 bp), forward: 5′-ACATAGCTGTGATGAGCAAT-3′ and reverse: 5′-AGTCTGGTGGACCCTCT-3′; and β-actin (281 bp), forward: 5′-GAGAGGGAAATCGTGCGTGAC-3′ and reverse: 5′-CATCTGCTGGAAGGTGGACAA-3′. Amplifications were performed with the following cyclic profile: the PCR mixture was denatured at 94 °C for 5 minutes, followed by 35 cycles (25 cycles for β-actin) of denaturation at 94 °C for 30 seconds, annealing at 55 °C for 20 seconds (A20), 60 °C for 20 seconds (TNF-α), 55 °C for 20 seconds (P65), or 57 °C for 30 seconds (β-actin), and extension at 72 °C for 20 seconds followed by a final extension step at 72 °C for 5 minutes.

### Western blot analysis

Cells were lysed with cell lysis buffer (Cell Signaling Technology, Beverly, MA, USA) supplemented with protease inhibitor. The intracellular or nuclear proteins were separated by 10% sodium dodecyl sulfate–polyacrylamide gel electrophoresis (SDS-PAGE) (Daiichi Pure Chemicals, Tokyo, Japan) and transferred onto polyvinylidenefluoride (PVDF) membranes (Millipore, Bedford, MA, USA). After subsequent blocking with skim milk, the membranes were incubated with primary antibodies (against A20, TNF-α, P65, and β-actin, all Santa Cruz Biotechnology, Santa Cruz, CA, USA) separately overnight at 4 °C. The secondary antibody was horseradish peroxidase (HRP)-conjugated rabbit anti mouse immunoglobulin (Ig). The immunoreactive proteins were detected by an enhanced chemiluminescent HRP substrate (Millipore) and exposed to Kodak X-Omat Film.

### Liver biochemistry markers and histopathological analysis

Serum levels of alanine aminotransferase (ALT), aspartate aminotransferase (AST), and total bilirubin (TBIL) were determined using a biochemical automatic analyzer Vitros750 (Johnson & Johnson, Rochester, NY, USA). The liver tissues harvested 6 hours after LPS/D-GalN injection were embedded in paraffin, and sections were stained with hematoxylin and eosin (H&E) for histological evaluation.

### Statistical analyses

Quantitative data are presented as means ± standard deviations (SDs). Differences among multiple groups were examined by one-way analysis of variance (ANOVA) or the least significant difference (LSD) test. The survival rate of mice after injection of LPS/D-GalN was analyzed using the log-rank method. A value of P < 0.05 was considered statistically significant.

## Results

### Isolation and phenotypic characterization of CD11C^low^CD45RB^high^DCs

**Figure 1:**
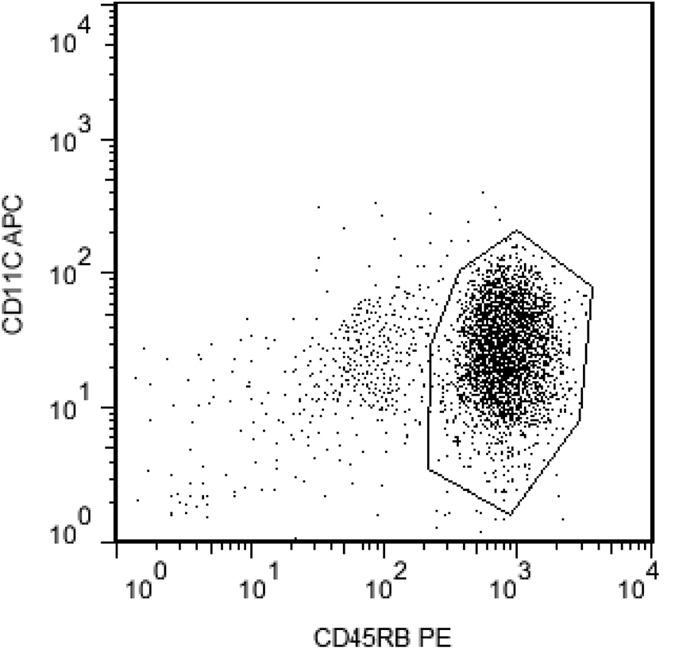
Magnetic-activated cell sorting of CD11C^low^CD45RB^high^ DCs. Splenic DCs were enriched and isolated from BALB/c mice, and CD11C^low^CD45RB^high^ DCs were collected by magnetic activated cell sorting. The purity of collected CD11C^low^CD45RB^high^ DCs was 90%.

The purity of isolated CD11C^low^CD45RB^high^ DCs from spleens of normal mice and mice with ET reached 90% as shown by MACS (Fig. 1). Moreover, substantially more splenic CD11C^low^CD45RB^high^ DCs (about 1.20 × 10^6^/mouse) were isolated from the mice pretreated with LPS than from normal mice (about 5.30 × 10^5^/mouse).

**Figure 2:**
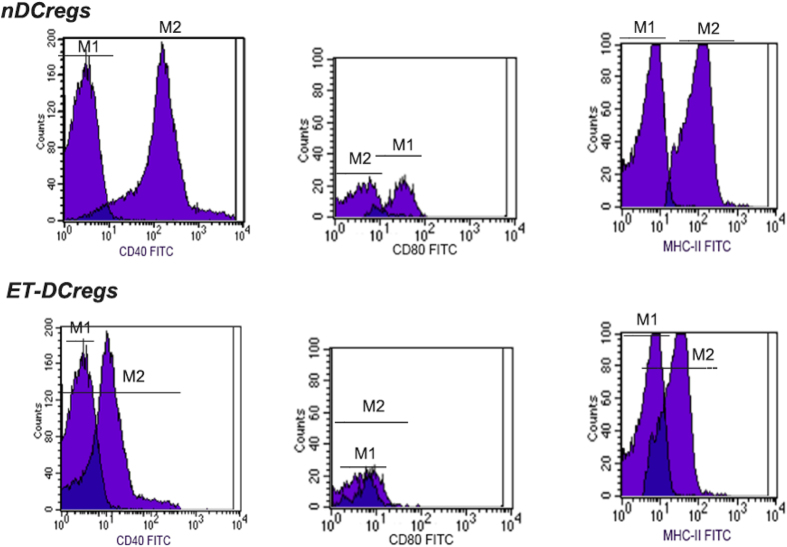
The effect of LPS pretreatment on expression of MHC-II and costimulatory molecules on splenic CD11C^low^CD45RB^high^ DCs. The M1 area represents the population and fluorescein isothiocyanate (FITC) staining with isotype-matched control IgG, while the M2 area represents CD11C^low^CD45RB^high^ DCs and FITC staining with respective antibodies. A shows splenic CD11C^low^CD45RB^high^ DCs derived from normal mice and the first to third columns indicate relative expression levels of 45.6% for CD40, 47.6% for CD80, and 41.2% for MHC-II, respectively. B shows splenic CD11C^low^CD45RB^high^ DCs from mice pretreated with LPS and three individual graphs from left to right indicate relative expression levels of 31.8% for CD40, 21.7% for CD80, and 28.7% MHC-II, respectively. Data from one of four representative experiments are shown.

The expression of costimulatory molecules CD40, CD80, and MHC-II was further examined on MACS-sorted CD11C^low^CD45RB^high^ DCs. As shown in Fig. 2, relatively low levels of MHC-II and moderate levels of CD40 and CD80 s were detected on splenic CD11C^low^CD45RB^high^ DCs from LPS-treated mice, as compared to their normal counterparts. The ability of CD11C^low^CD45RB^high^ DCs to suppress allogenic antigen-specific CD4+ T-cell proliferation was also investigated. When CD11C^low^CD45RB^high^ DCs were co-cultured with CD4+ T cells at various ratios, including 1:10, 1:50, and 1:100, for 24 hours, as shown Table 1, reduced CD4+ T-cell proliferation was observed with CD11C^low^CD45RB^high^ DCs from LPS-treated mice, and the culture medium from the same CD11C^low^CD45RB^high^ DCs contained a higher concentration of IL-10 and lower concentration of IL-12, indicating a tilled differentiation to Th1 cells, compared to that from CD11C^low^CD45RB^high^ DCs from normal mice.

### Effect of CD11C^low^CD45RB^high^ DCs on experimental ALF

**Figure 3:**
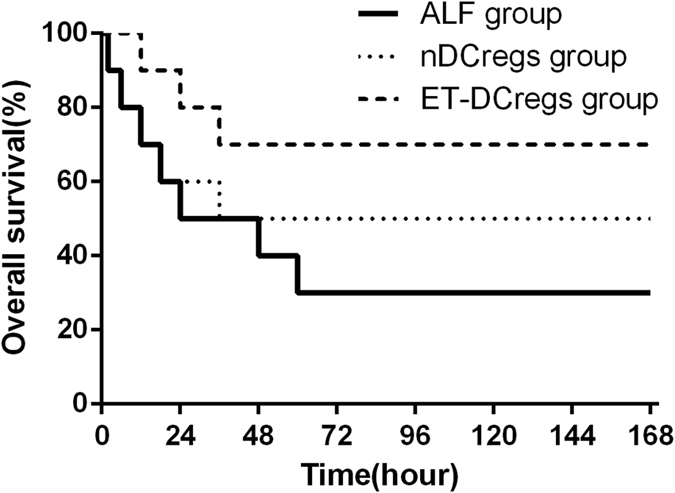
Effects of DCreg injection on survival rate of ALF mice. After injection of D-GalN/ LPS, significant mortality was observed in the ALF group. However, mortality was significantly reduced in the DCreg injection group, especially in the ET-DCregs group.

**Figure 4:**
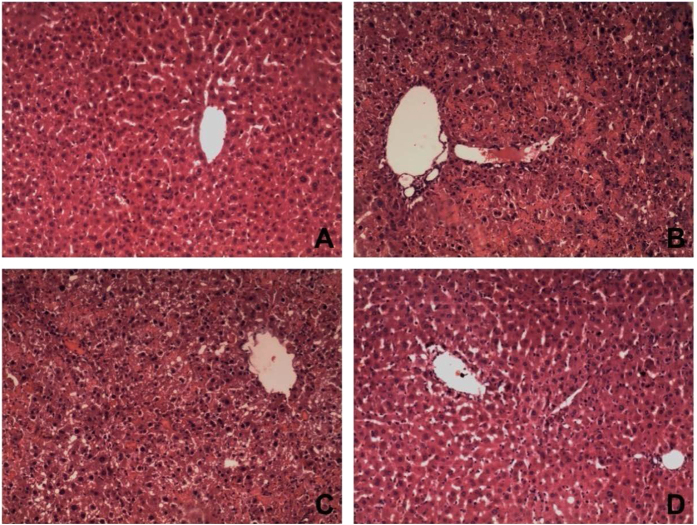
Liver histology (HE-stained sections) from control (**A**), ALF (**B**), nDCregs (**C**), and ET-DCregs (**D**) groups (original magnification, ×200). The hepatic cords and lobules were disordered, and a large number of inflammatory cells had infiltrated the parenchyma in the ALF group compared to the results in normal rats. Although reduced liver injury was observed in the nDCregs group, the inflammatory infiltration was still significant. In the ET-DCregs group, liver injury was significantly mitigated and the hepatic lobule structure was intact.

**Figure 5:**
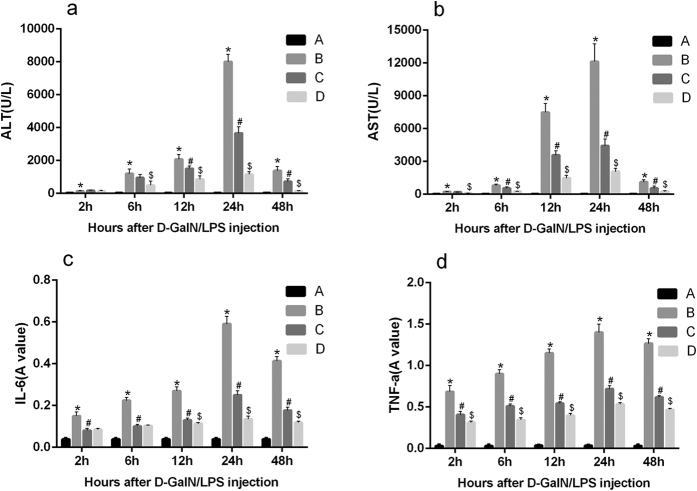
Changes in the serum levels of ALT, AST, and cytokines after D-GalN/LPS injection. Serum concentration kinetics of ALT (**a**), AST (**b**), IL-6 (**c**), and TNF-α (**d**). Data represent the mean ± SD from 6 rats at each time point. A: control group; B: ALF group; C: ALF+nDCregs group; and D: ALT+ET-DCregs group. Values in C and D are relative immunofluorescence absorbance values. *p < 0.05 versus control group, ^#^p < 0.01 versus ALF group and ^$^p < 0.01 versus nDCregs group.

To investigate the effect of DCregs against ALF, mice were treated with ex vivo purified CD11C^low^CD45RB^high^ DCs after injection of D-GalN/LPS. The 7-day survival was monitored (Fig. 3). The survival rates of mice were 30% (3/10) in ALF group and 50% (5/10) in ALF+nDCregs group, while the highest survival rate (70%, 7/10) was observed in the ALF+ET-DCregs group. The administration of special dose D-GalN/LPS may cause severe hepatic damage, high levels of endoxemia, excessive inflammation, and ultimately death. As shown in Fig. 4, severe hepatocyte death, inflammatory cell infiltration, and disordered hepatic lobules were observed in the liver of ALP mice (Fig. 4B). However, there were significant differences in pathological changes in the liver between the ALF+nDCregs and ALF+ET-DCregs groups. In the ALF+nDCregs group, the hepatic cords and lobules were disordered, and inflammatory cells had infiltrated (Fig. 4C). In the ALF+ET-DCregs group, the liver injuries were much less prominent (Fig. 4D), as evidenced by the intact structure of hepatic cords and lobules despite the presence of swelling in hepatocytes and inflammatory cell infiltration. In addition, as shown in Fig. 5a and 5b, the serum levels of ALT and AST were significantly elevated after injection of D-GalN/LPS and reached a peak at 24 hours in the ALF group, whereas lower levels of ALT and AST were observed in the therapy groups. Further, the levels of ALT and AST in the ALF+ ET-DCregs group were significantly lower than those in the ALF+nDCregs group at every time point.

### Possible mechanisms by which CD11C^low^CD45RB^high^ DCs attenuate experimental ALF

**Figure 6:**
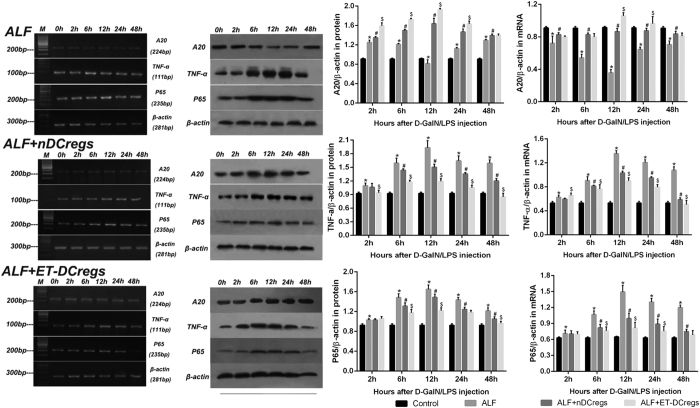
Effects of splenic CD11C^low^CD45RB^high^ DC transfer on mRNA and protein expression of TNF-α, NF-κB, and A20 in the liver. The right column shows the mRNA expression level, and the left column displays protein expression level. The top histograms represent A20, the middle histograms represent TNF-α, and the bottom histograms represent P65. The data are presented as mean ± SD from six mice. *p < 0.05 versus control group, ^#^p < 0.01 versus ALF group and ^$^p < 0.01 versus nDCregs group.

To gain insight into the mechanisms that mediate the protective effect of DCregs against ALF, the effects of DCregs on D-GalN/LPS-induced proinflammatory production in mice were examined. As shown in Fig. 5c,d, D-GalN/LPS treatment transiently elevated levels of serum TNF-α and IL-6 in ALF mice, whereas nDCreg treatment lowered levels of two cytokines in the serum. Moreover, more potent suppression of D-GalN/LPS-induced serum TNF-α and IL-6 expression was detected after ET-DCreg treatment than after nDCreg treatment. The intracellular inflammatory signaling pathway was further investigated. As shown in Fig. 6, a significant increase in P65 expression was observed in the ALF group compared to that in the control group. Nuclear factor (NF)-κB expression was suppressed with DCreg treatment, and it was more prominently inhibited with ET-DCreg treatment. Moreover, A20 expression was reduced after injection of D-GalN/LPS in the ALF group, whereas it was increased with DCreg treatment. A further increase was noted with ET-DCreg treatment.

## Discussion

DCs are professional APCs, and antigen presentation is an early and essential step for initiating a specific immune response^11^. For example, DCs loaded with tumor-associated antigens (TAAs) ex vivo induce antigen-specific immune responses in both *in vitro* and *in vivo* animal models^12^.

In addition to inducing the specific immunity, DCs can also induce immunological tolerance. DCregs are a subset of DCs that induce Tregs or directly inhibit T-cell proliferation and/or induce T-cell anergy. Regulation of T cells by DCregs is mediated by costimulatory molecules CD40/CD80 and MHC-II expressed on the cell surface. Our results indicated that the surface expression of CD40, CD80, and MHC-II by DCregs in the spleen was down-regulated after exposure to LPS. This finding may have therapeutic implications^13^,^14^,^15^. CD40 is one of the most important costimulatory molecules on the DC surface. The CD40/CD40 ligand (CD40L) system can promote cytokine secretion, up-regulate the expression of adhesion molecules and costimulatory molecules, and enhance the CTL (cytotoxic T lymphocytes) effect of CD8^+^ T cells^16^,^17^. Thus, reduced CD40 expression could reduce cytokine production and secretion and suppress CTL function. In addition to the impact on costimulatory molecules, IL-10 production was increased, and IL-12 expression was inhibited in DCregs. Altered expression of these cytokines could tilt T-cell differentiation toward Th1 or Th2 dominance. Our results showed that the splenic DCs derived from mice after LPS pretreatment showed a subdued ability to present antigens.

The liver is also an important immune organ, and full liver function is required for balanced and well-functioned immunity. It is expected that the immunity is under stress and severely disturbed upon ALF caused by severe liver injury. D-GalN/LPS injection induces ALF in mice, and the clinical course resembles that of human ALF^1^. D-GalN can induce liver injury mainly by depleting uracil nucleotides and triggering apoptosis and necrosis of hepatocytes^18^, and inclusion of LPS can further aggravate liver injury^19^. The major signaling pathway that leads to liver injury after LPS/GalN treatment is TLR4-stimulated production of TNFa in Kupffer cells and other macrophages, and TNF binds to TNFR on hepatocytes that initiates apoptosis (pro-survival gene expression is suppressed by the nucleotide depletion caused by GalN). In addition, special antigens could be presented to T cells in the circulation by DCs and these T cells thereby stimulated NF-kB activation, increased TNF-α production. which binds to TNFR on hepatocytes to cause apoptosis in mice with LPS/GalN treatment. In our study, ET-DCs injection could theoretically induce generation of regulatory T cells incapable of stimulating T cells and T cell apoptosis. Consequently, both decreased cytokines expression (especially TNFa) and alleviated liver injury were detected in mice with ET-DCs treatment. The mechanism might partially show how these regulatory dendritic cells protect mice against LPS/GalN treatment.

Inflammatory cytokines may augment pathological changes and facilitate the progression of liver failure^20^. In our study, we found that the expression levels of TNF-α and IL-6 were significantly increased after D-GalN/LPS injection. However, treatment of mice with nDCregs significantly suppressed these inflammatory cytokine levels. Moreover, DCregs from mice with ET achieved more potent suppression of liver inflammation than did DCregs from normal mice. Immunotherapy using antigen-pulsed DCregs has shown a beneficial effect in experimental allergic asthma^21^. Antigen-pulsed mature DCs can suppress TH2-mediated allergic diseases through an IL-12–mediated TH1-skewing immune response^22^. In this study, T cell-induced cytokine secretion was regulated by ET-DCregs, and the immunotherapeutic protection offered by DCreg treatment may result from the reduced inflammation mediated by DCs. Instead of entering the circulation to affect T cells, our results suggested that the protective effect of i.p. injected DCregs in experimental ALF locally produced IL-10, leading to the inhibition of CD4+T-cell function as well as pro-inflammatory activation in draining lymphoid areas, such as retroperitoneal lymph nodes. However, additional mechanisms by which DCregs regulate CD4+T-cell function as well as pro-inflammatory activation remain unclear.

IL-10 produced by the dendritic cells can inhibit NF kB activation and attenuate TNFa production, which explained how the DCregs deliver the protective effect. Moreover, Santucci *et al*. in 1996 showed that injection of IL-10 itself could reduce plasma TNF-α concentration, LPS/D-GalN-induced hepatic injury and lethality in mice^23^. To further clarify the role of IL-10, a complementary experiment is underway to explore their inhibitory properties and abilities in ameliorating D-GalN/LPS-induced liver injury by siRNA silencing of IL-10 in ET-DCregs. Taken together, our results suggested that LPS pretreatment might direct remodeling of cellular gene expression in DCregs, which may impact their antigen-presenting function.

NF-κB is involved in many biological functions including the immune response and apoptosis. Expression of the inflammatory cytokines TNF-α and IL-6 is rapidly induced in response to stimulation by antigens, infection, and stress, and are transcriptionally regulated by NF-κB^24^. Our previous study suggested that D-GalN/LPS-induced liver apoptosis was mainly mediated by TNF-α, and that TNF-α levels closely correlated with rat survival rates^25^,^26^. The results in the present study suggested that the protective effects of DCreg therapy against ALF is probably mediated via the reduced expression of inflammatory cytokines. In addition, NF-κB also regulates the transcription of a number of cellular genes associated with innate immune responses. Activation of NF-κB is tightly controlled by a group of inhibitory proteins. Because DCregs derived from endotoxin-tolerant mice could significantly increase IL-10 production and IL-10 can inhibit NF-κB activation^27^, down-regulation of NF-κB activation may lead to inhibition of TNF-α and IL-6 expression in DCreg-treated mice.

In conclusion, Pretreatment of mice with LPS significantly alters the functionality of splenic DCs, and transfer of splenic DCs from endotoxin-tolerant mice could mitigate D-GalN/LPS-induced acute liver injury via suppression of NF-κB activation and down-regulation of inflammatory cytokines.

## Additional Information

**How to cite this article**: Zhang, S.-N. *et al*. Splenic CD11c^low^CD45RB^high^ dendritic cells derived from endotoxin-tolerant mice attenuate experimental acute liver failure. *Sci. Rep.* **6**, 33206; doi: 10.1038/srep33206 (2016).

## Figures and Tables

**Table 1:** The proliferation ratio and the concentration of IL-10 and IL-12 in different DCregs/T cell groups.

Group	Proliferation ratio (%)	IL-10 (pg/mL)	IL-12 (pg/mL)
Control	80.23 ± 2.47	150.37 ± 22.25	230.37 ± 23.28
nDCregs/T cells 1:10	75.60 ± 1.66*	750.49 ± 142.23*	170.87 ± 22.73*
1:50	80.54 ± 2.35	720.32 ± 110.15*	202.32 ± 30.15*
1:100	84.31 ± 1.63	680.69 ± 95.32*	223.69 ± 31.15*
ET-DCregs/T cells 1:10	63.21 ± 1.21*^#^	1850.73 ± 137.46*^#^	142.69 ± 13.26*^#^
1:50	68.48 ± 2.26*^#^	1632.63 ± 124.35*^#^	164.85 ± 14.63*^#^
1:100	72.53 ± 1.73*^#^	1277.46 ± 124.86*^#^	182.38 ± 13.32*^#^
